# Betulinic acid is associated with miR-21 modulation, apoptosis and redox changes in breast cancer cells: an in vitro and in silico study

**DOI:** 10.1038/s41598-026-51156-z

**Published:** 2026-05-16

**Authors:** Mohammad A. Mahmoud, Hagar Abdo, Mariam Mekky, Tasneem Salem, Sara Mabrouk, Basmala Khaled

**Affiliations:** 1 Department of Chemistry, Faculty of Science, Kafrelsheikh University, Kafrelsheikh, 33516 Egypt; 2 Bella Secondary School For Girls, Kafrelsheikh, Egypt

**Keywords:** Betulinic acid, Doxorubicin, miRNA, Breast cancer, Molecular docking, Biochemistry, Cancer, Cell biology, Drug discovery, Molecular biology, Oncology

## Abstract

Betulinic acid is defined as a hydrophobic pentacyclic triterpenoid primarily found in the bark of Betula alba, known for its anticancer activity through mechanisms such as upregulating proapoptotic proteins, modulating NF-kB, and inhibiting topoisomerase I. The current study aimed to explore the anticancer potential effect, molecular targets, and association with miR-21 modulation after BA treatment and its combination with doxorubicin against human triple-negative breast cancer (MDA-MB-231) cells. We determined drug cytotoxicity by MTT assay, and death mechanism by flow cytometry. Besides, the potential effect of BA and DOX treatment on downstream effect of miR-21 on HIF1A, PDCD4, PTEN and SMAD7 expression levels. Finally, a molecular docking study was performed to determine molecular targets, binding affinity, and mode of interactions of BA and DOX with HIF1A, PDCD4, PTEN and SMAD7. Collectively, our data showed that treatment with BA and/or DOX have significantly describing observed differences under the tested conditions associated with increasing apoptosis, downregulation of miR-21, HIF1A and SMAD7 expression and upregulation of PDCD4 and PTEN. Finally, the molecular docking study suggested potential interactions between BA and DOX with PTEN and PDCD4, modulating their activities leading to growth arrest and cell death. In conclusion, this study reported BA as potential antiproliferative compound with modulation of miR-21 expression and identified molecular targets involved in its action.

## Introduction

Cancer was initially identified as a tumor, a swelling mass of tissue^1^. Cancer accounts for nearly one in six fatalities (16.8%) and one in four deaths (22.8%) from non-communicable diseases (NCDs) globally, making it a significant social, public health, and economic issue in the twenty-first century. Cancer is still one of the biggest causes of death, with almost 20 million new cases reported worldwide in 2022^2^. According to the most recent GLOBOCAN 2022 estimates, over 53 million people survive within five years of receiving a cancer diagnosis, while the disease causes 9.7 million deaths annually^3^. The most common malignancies to be diagnosed are lung^4^, breast^5^, prostate^6^, and colorectal^7^, which are also among the top causes of cancer-related mortality globally^8^.

Breast cancer (BC), which accounts for 11.7% of all cancer cases, is a major source of morbidity and mortality in women^9^. Breast cancer is the most prevalent disease diagnosed worldwide (2.3 million new cases, 11.5% of all cancer cases) and the primary cause of cancer incidence in 159 countries. It is the top cause of cancer death in women and the fourth most common cause of cancer mortality overall, accounting for about 666,000 deaths in 2022^10^. Breast cancer is a complicated tumor with a number of unique subtypes that differ in their molecular and clinical features. Triple-negative breast cancer (TNBC) is one of these subtypes that is prevalent and presents serious therapeutic and prognostic problems. TNBC, which makes up 15–20% of all breast cancers, is characterized by the lack of expression of the estrogen receptor (ER), progesterone receptor (PR), and human epidermal growth factor receptor 2 (HER2)^11^. TNBC is a biologically varied collection of tumors with a range of transcriptomic, genomic, and epigenetic characteristics, which can lead to differences in clinical outcomes and therapeutic responsiveness^12^.

MicroRNAs (miRNAs) are known as small noncoding RNAs that are crucial for controlling posttranscriptional gene expression. MicroRNA-21 (miR-21), one of the first oncogenic miRNAs identified, has been emphasized for its crucial involvement in malignancies. The primary miRNA involved in the invasion process of breast cancer, miR-21, promotes tumor growth and metastasis. MiR-21 acts as an oncogene by attacking several tumor/metastasis suppressor genes. MiR-21 can serve as a prognostic marker in breast cancer since its elevated expression has been linked to lymph node metastasis, advanced tumor levels, and a poor prognosis. Thus, regulating important carcinogenic pathways in TNBC may be possible by focusing on miR-21^13–15^.

Chemotherapy is designed to prevent invasion and metastasis by preventing tumor growth and cell proliferation. By interfering with the production of DNA, RNA, or proteins or by inhibiting the proper functioning of the preformed molecule, traditional chemotherapeutic drugs mainly influence the macromolecular synthesis or function of neoplastic cells. However, because chemotherapy affects healthy cells, this has harmful repercussions^16^.

Anthracyclines, especially doxorubicin (DOX), are frequently used as part of therapy regimens for breast cancer. One of the most used chemotherapeutic medications is DOX, which can stop cell division and cause p53-dependent apoptosis in malignant tissue. However, there are both immediate and long-term consequences to its use, including mild to severe toxicities. DOX can cause indirect neurotoxicity through neuroinflammatory processes, which may result in cognitive problems even if it cannot pass through the blood-brain barrier^17^. One of the more common and potentially dangerous side effects of cancer treatment is cardiotoxicity that have an impact on the quality of life and mortality^18^. Cardiotoxicity is characterized by a decrease in the left ventricular ejection fraction (LVEF). In this regard, using natural substances may be a viable strategy to enhance therapeutic results, either by increasing anticancer activity or possibly lowering treatment-related toxicity.

The anticancer properties of natural substances produced from microbes, plants, and other living species have been the subject of extensive research in recent decades. Many natural items are used by cancer patients as part of a complementary and integrative approach to their healthcare, and some commonly used anticancer medications come from natural sources^19^.

A promising anticancer therapeutic candidate, betulinic acid (BA) is a pentacyclic triterpenoid of the lupane type that has been extensively researched for its ability to decrease tumor growth and prevent the proliferation of cancer cells^20^. Through a number of mechanisms, such as topoisomerase inhibition, reactive oxygen species (ROS) production, apoptosis via the mitochondrial system, and suppression of the NF-kB signaling pathway, sp transcription factors, and estrogen receptors, it demonstrates potent anti-breast cancer efficacy. By blocking matrix metalloproteinases 2 and 9 (MMP-2 and MMP-9), as well as vascular endothelial growth factors and receptors, BA also exhibits anti-angiogenic and anti-metastatic properties^21^. The taxonomic hierarchy of Plants Containing BA is shown in Table 1.

**Table 1:** Taxonomic hierarchy of Plants Containing Betulinic Acid.

Plant name	Scientific Name	Kingdom	Superkingdom	Subkingdom	Division	Class	Subclass	Order	Family	Species
Silver Birch	Betula pendula	Plantae	Eukaryota	Viridiplantae	Tracheophyta	Magnoliopsida	Rosidae	Fagales	Betulaceae	Betula
White Birch	Betula alba	Plantae	Eukaryota	Viridiplantae	Tracheophyta	Magnoliopsida	Rosidae	Fagales	Betulaceae	Betula
Guava	Psidium guajava	Plantae	Eukaryota	Viridiplantae	Tracheophyta	Magnoliopsida	Rosidae	Myrtales	Myrtaceae	Psidium
Baobab	Adansonia digitata	Plantae	Eukaryota	Viridiplantae	Tracheophyta	Magnoliopsida	Rosidae	Malvales	Malvaceae	Adansonia
Henna	Lawsonia inermis	Plantae	Eukaryota	Viridiplantae	Tracheophyta	Magnoliopsida	Rosidae	Myrtales	Lythraceae	Lawsonia
Mango	Mangifera indica	Plantae	Eukaryota	Viridiplantae	Tracheophyta	Magnoliopsida	Rosidae	Sapindales	Anacardiaceae	Mangifera
Apple	Malus domestica	Plantae	Eukaryota	Viridiplantae	Tracheophyta	Magnoliopsida	Rosidae	Rosales	Rosaceae	Malus
Hawthorn	Crataegus oxyacantha	Plantae	Eukaryota	Viridiplantae	Tracheophyta	Magnoliopsida	Rosidae	Rosales	Rosaceae	Crataegus
Eucalyptus	Eucalyptus globulus	Plantae	Eukaryota	Viridiplantae	Tracheophyta	Magnoliopsida	Rosidae	Myrtales	Myrtaceae	Eucalyptus
African Cherry	Prunus africana	Plantae	Eukaryota	Viridiplantae	Tracheophyta	Magnoliopsida	Rosidae	Rosales	Rosaceae	Prunus
Silver Birch	Betula pendula	Plantae	Eukaryota	Viridiplantae	Tracheophyta	Magnoliopsida	Rosidae	Fagales	Betulaceae	Betula

DOX has well-known anticancer potential and also BA as stated in the CTD database (http://ctdbase.org/). DOX is used as a therapy for about 2933 cancer clinical trials and BA as recognized in the clinical trials database (https://clinicaltrials.gov/).

Thus, the goal of the current study was to examine the anticancer effects of BA on triple-negative breast cancer (MDA-MB-231) cells, specifically focusing on changes in oxidative stress, apoptosis, and miR-21 modulation. As well as to evaluate its combined effects with DOX under the tested conditions. Furthermore, molecular docking analysis was performed to explore potential interactions with selected targets.

## Materials and methods

### Chemicals and reagents

BA was obtained from Adamas-beta Co., Ltd. (Shanghai, China) and DOX was purchased from Sigma-Aldrich Chemical Co. (St. Louis, Missouri, USA). All cell culture materials were obtained from Gibco (New York, New York, USA).

### Cell lines

Human breast cancer MDA-MB-231 cells were supplied from American Type Culture Collection (ATCC). Cells were cultured in a complete DMEM medium and incubated at 37 °C in an atmosphere containing 5% CO_2_.

### Cytotoxicity assay

Cell viability was measured by MTT as previously described^22^. Briefly, MDA-MB-231 Cells were cultured at 15 × 10^3^ per well in a 96-well plate with 100 µl of complete fresh medium for 24 h before treatment with different concentrations of BA and DOX (6.25–100 µM) for 48 h. After incubation, 20 µL (5 mg/mL) of MTT reagent (SERVA Electrophoresis GmbH, Heidelberg, Germany; cat. no. 20395.01) was added to each well and incubated for 4 h at 37 °C. After that, the formazan crystals formed were dissolved in 100 µL of dimethyl sulfoxide (DMSO). The optical density was measured at 570 nm using an ELISA plate reader. The IC50 was calculated by nonlinear regression analysis of the dose-response curve.

### Cell line treatment

Cells were treated with the predetermined calculated IC_50_ values for all cellular and molecular analysis for each compound. Both cell lines were treated as following: culture media or 0.1% DMSO controls; single treatment with either BA or DOX and double treatment with BA + DOX. Treatment was performed 48 h before the respective analysis, and experiments were repeated at least three times.

### Assessment of apoptosis and necrosis by Annexin V-FITC/PI staining

The manufacturer’s instructions were followed while using the FITC-Annexin V Apoptosis Detection Kit (BD Bioscience, Heidelberg, Germany; cat. no. 556547) as previously described^23^. To put it briefly, collected and floating cells were combined, twice cleaned with cold phosphate buffered saline (PBS), and then resuspended in binding buffer at a final density of 10^6^ cells/ml. 100 µL of the cell solution containing 10^5^ cells was mixed with 5 µl of FITC-annexin and 5 µl of PI. After gently vortexing the cell suspension, it was incubated for 15 min at room temperature in the dark. The cells were then subjected to flow cytometry using FACS Calibur (BD Bioscience, Heidelberg, Germany) and Cellquest Pro software (BD Bioscience) after 400 µl of binding buffer was added.

### RNA extraction, cDNA synthesis, and quantitative real-time PCR

The mRNA levels of Hypoxia-Inducible Factor 1-alpha (HIF-1α), Programmed Cell Death 4 (PDCD4), Phosphatase and tensin homolog (PTEN), SMAD7 genes, and miRNA-21 were assessed by qRT-PCR. Total RNA was firstly isolated using the miRNeasy Mini Kit (Qiagen, Germany; cat. no. 217004) and reverse‑transcribed to cDNA using the QuantiTect Reverse Transcription kit (Qiagen, Germany; cat. no. 205311) as previously described^24^. Briefly, after lysing the cells, spin columns were used to purify the total RNA, which was then washed and eluted, then, the reaction mixture for cDNA was incubated for 30 min at 42 °C and then inactivated for 3 min at 95 °C. Second, qRT-PCR was performed using the qPCR Master Mix kit (Enzynomics, Korea; cat. no. RT500S) in a 20 µL reaction volume that included the cDNA template, forward and reverse primers, and SYBR Green mix. The qRT-PCR cycles consisted of 10 min at 95 °C, 40 cycles of denaturation at 95 °C for 10 s, annealing at 60 °C for 15 s and extension at 72 °C for 15 s. The primers for *HIF-1α*,* PDCD4*,* PTEN*,* SMAD7*, *miRNA-21*, *β-actin*, and *U6* genes are listed in Table 2. The relative expression of *HIF-1α*,* PDCD4*,* PTEN and SMAD7* were calculated by the comparative 2^−ΔΔCt^ method^25^ using the endogenous *β-actin* as a housekeeping gene, while *miRNA-21* was calculated using the *U6* gene as an endogenous control.

**Table 2:** Primers used in real-time PCR amplification.

Target gene	Forward sequence (5′ to 3′)	Reverse sequence (5′ to 3′)
PDCD4	TTCTACAAACCGCTCCAGCAC	GGCAGCAATCTGTCAATCACC
HIF1α	CCTCTAACCTGCTGCTCATTGG	GTAGAGGCACTCGAACTGATCC
PTEN	ACCCACCACAGCTAGAACTT	GGGAATAGTTACTCCCTTTTTGTC
SMAD7	CCTTCCTCCGCTGAAACAG	CCCACTCTCGTCTTCTCCT
Β-actin	TCCTCCCTGGAGAAGAGCTA	CGTGGATGCCACAGGACT
miR-21	AGTTTTCTTGCCGTTCTGTAAGT	GTCATGAAGACTATCCCCATTTC
U6	ATTGGAACGATACAGAGAAGATT	GGAACGCTTCACGAATTTG

### Activity of SOD and CAT

Superoxide Dismutase (SOD) and catalase activities were measured using available commercially kits (Bio-diagnostic, Dokki, Giza, Egypt; cat. no. SD 25 21 and CA 25 17, respectively), according to the producer’s instruction. In briefly, the cells were lysed using lysis buffer provided then cells were centrifuged at 12,000 rpm for 10 min at 4 °C, the supernatant was aspirated and the pellet was discarded. SOD activity was determined by its capacity to prevent the reduction of nitroblue tetrazolium, and the absorbance was measured at 560 nm. The rate of hydrogen peroxide (H_2_O_2_) breakdown was used to determine catalase activity, and the absorbance was measured at 240 nm^26^.

### Molecular docking

To perform molecular docking of BA and DOX against HIF1A (target site PDB ID: 8HE3), PDCD4 (target site PDB ID: 2RG8), PTEN (target site PDB ID: 1D5R) and SMAD7 (target site PDB ID: 2DJY), we firstly downloaded from RCSB PDB database (https://www.rcsb.org/*)* and prepared by BIOVIA Discovery Studio Visualizer software (https://discover.3ds.com/discovery-studio-visualizer-download*)*^27,28^. In addition, the 3D structures of BA and DOX were obtained from the PubChem database (https://pubchem.ncbi.nlm.nih.gov/*).* The binding free energy, binding affinity (p*Ki*), and the ligand efficiency of BA and DOX against prepared HIF1A (8HE3), PDCD4 (2RG8), PTEN (1D5R) and SMAD7 (2DJY) were determined using InstaDock software^29^. Finally, BIOVIA Discovery Studio Visualizer software did the visualization of target-ligand interaction.

### Statistical analysis

Statistical analysis was performed using Graphpad prism 8.0.1 (https://www.graphpad.com/*).* Data were presented as mean ± SEM. One- way ANOVA and Tukey’s multiple comparison test were used to compare group differences, *p <* 0.05 was deemed to show statistical significance. All tests were performed at least three independent experiments (*n* = 3) each performed in triplicate.

## Results

### Effect of BA and DOX on proliferation of breast cancer cells

**Fig. 1:**
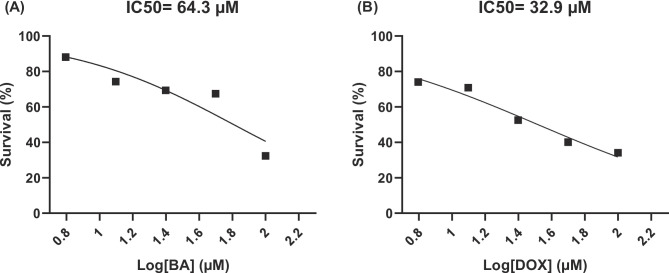
Dose response curves of (**A**) betulinic acid (BA) and (**B**) doxorubicin (DOX) on the proliferation of MDA-MB-231 cells: Cells were exposed to different concentrations of each compound (6.25–100 µM) for 48 h and cell viability was determined by MTT assay. Data are expressed as the mean ± SEM of three independent experiments (*n* = 3) each performed in triplicate. Curves were generated using nonlinear regression analysis with Log concentrations. Ic50 values were calculated from the fitted curves.

The antiproliferative effect of BA and DOX was investigated in MDA-MB-231 cells after 48 h treatments using the MTT assay. Dose–response curves were used to determine the IC50 values for BA and DOX. A dose-dependent growth inhibition was observed in BA and DOX treated cells compared to control cells. The IC50 values for BA and DOX were 64.3 and 32.9 µM, respectively (Fig. 1).

**Fig. 2:**
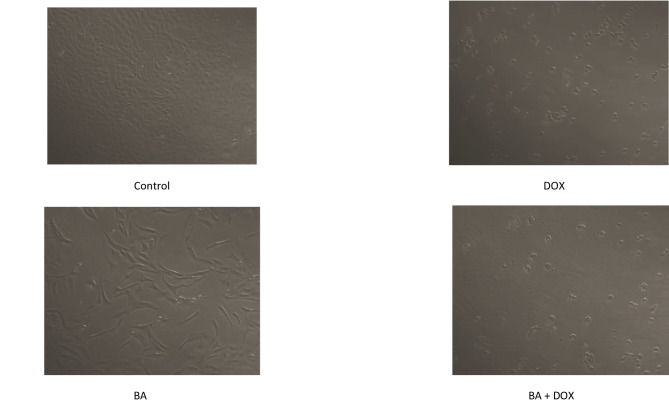
Representative photographs showing morphological changes in MDA-MB-231 cells after treatment with IC50s of single or combined betulinic acid (BA) and doxorubicin (DOX) (64.3 and 32.9 µM, respectively). Cells were examined and photographed under light microscope 20x.

After being exposed to BA and DOX for 48 h, MDA-MB-231 cells underwent a microscopic examination that revealed a significant decrease in cell numbers, decreased viability, and morphological changes, including loss of normal architecture, cell shrinkage that appeared round and wrinkled, condensation of cytoplasm, an increase in membrane roughness, and a loss of the ability to adhere to the surface of the culture plate. After treating MDA-MB-231 with BA + DOX, the greatest cytotoxicity was seen. Conversely, control cells proliferated effectively, achieving 100% confluency while adhering to the cell culture plate, and showed no discernible morphological change or death symptoms (Fig. 2).

### Assessment of apoptosis by flow cytometry

**Fig. 3:**
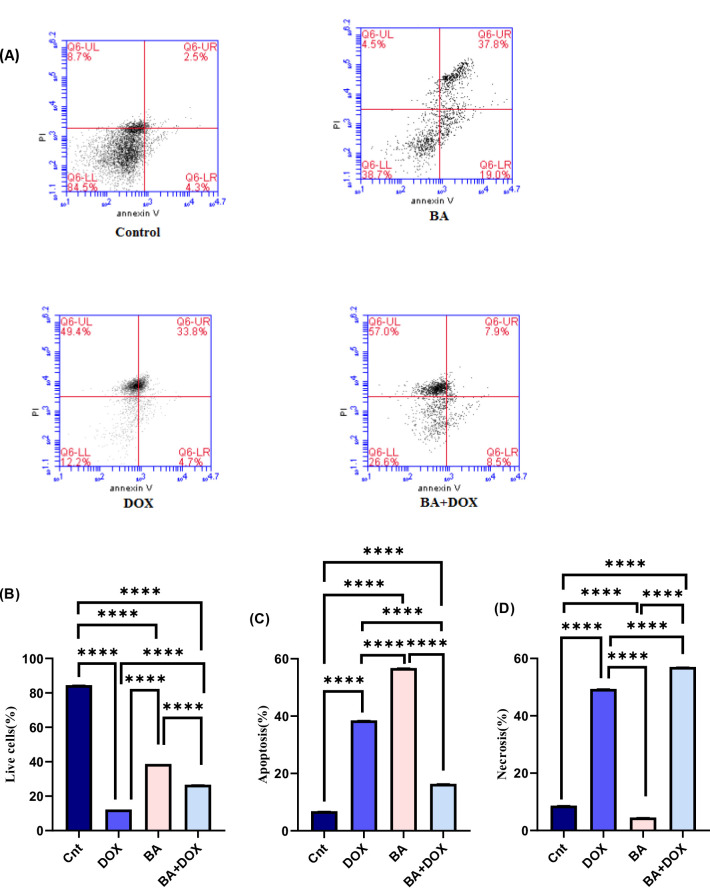
Assessment of cell death mechanism in MDA-MB-231 after betulinic acid (BA) and doxorubicin (DOX) alone and combination treatments with IC50s (64.3 and 32.9 µM, respectively) for 48 h. Cells were stained with annexin V-FITC and PI, analyzed by flow cytometry. Representative dot plots are shown (**A**), percentage of live cells, apoptotic (early + late) and necrotic cells (**B**, **C** and **D**). Data are expressed as the mean ± SEM of three independent experiments (*n* = 3) each performed in triplicate. Statistical analysis was performed using one-way ANOVA followed by Tukey’s post hoc test. Means within columns carrying **** are significantly different at (p ˂ 0.0001).

MDA-MB-231 cell line was treated with BA (64.3 µM) and/or DOX (32.9 µM) for 48 h to investigate drug effects on the percentages of viable and dead cells. As shown in Fig. 3, the treatment of BA + DOX was associated with more necrosis effect on the MDA-MB-231 cell line compared with the control (*P* < 0.0001) (Fig. 3D). The highest percentage of apoptotic cells were detected after treatment of MDA-MB-231 cells with BA (56.8% ± 0.034) (Fig. 3C). Flow cytometry analysis indicated that the highest cytotoxic effect showing the lowest number of viable cells was observed after treatment of MDA-MB-231 with DOX (12.2% ± 0.012) (Fig. 3B).

### Influence of BA and DOX on relative expression of HIF-1α, PDCD4, PTEN, SMAD7 and miRNA-21

**Fig. 4:**
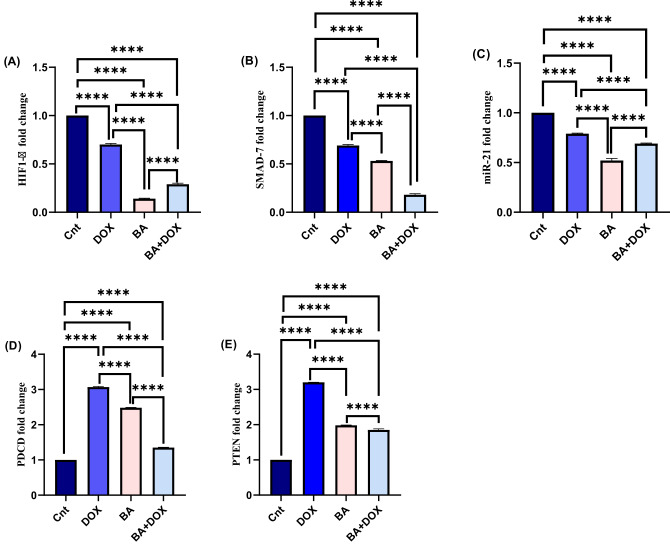
Real time-PCR analysis for studied genes [Hypoxia-Inducible Factor 1-alpha (HIF-1α), Programmed Cell Death 4 (PDCD4), Phosphatase and tensin homolog (PTEN), SMAD7, and miRNA-21] after betulinic acid (BA), and doxorubicin (DOX) treatments with IC50s (64.3 and 32.9 µM, respectively) for 48 h in MDA-MB-231 cells (**A**-**E**). Expression levels were normalized to *β-actin* for *HIF-1α*,* PDCD4*,* PTEN and SMAD7*and U6 for *miRNA-21*and calculated using 2^−ΔΔCt^ method. The data provided are mean ± SEM of three independent experiments (*n* = 3) each performed in triplicate. Statistical analysis was performed using one-way ANOVA followed by Tukey’s post hoc test. Means within columns carrying **** are significantly different at (p ˂ 0.0001).

Real time PCR was used to detect the relative expression of HIF-1α, PDCD4, PTEN, SMAD7 genes and miRNA-21 that reflects the changes in transcription levels of these genes in MDA-MB-231 cells after treatment with BA (64.3 µM) and/or DOX (32.9 µM) for 48 h in comparison to control. Our results revealed a significant (P ˂ 0.0001) upregulation of PDCD and PTEN gene expression following treatment with BA and DOX alone and in combination, with highest expression in the DOX group, as compared to non-treated group (Fig. 4D and E). On the other hand, the same treatments resulted in a significant (P ˂ 0.0001) downregulation of HIF-1α (approximately 3.4 and 1.4 folds) and SMAD7 (approximately 5.5 and 1.4 folds) genes in BA and DOX treated groups, with lowest expression in the BA group, as compared to non-treated group (Fig. 4A and B). Also, administration of BA and DOX showed a significant downregulation of miR-21 expression (approximately 1.45 and 1.27 folds), with the lowest expression was associated with BA treated group, as compared to non-treated group (Fig. 4C).

### Effect of BA and DOX on antioxidant enzymes

**Fig. 5:**
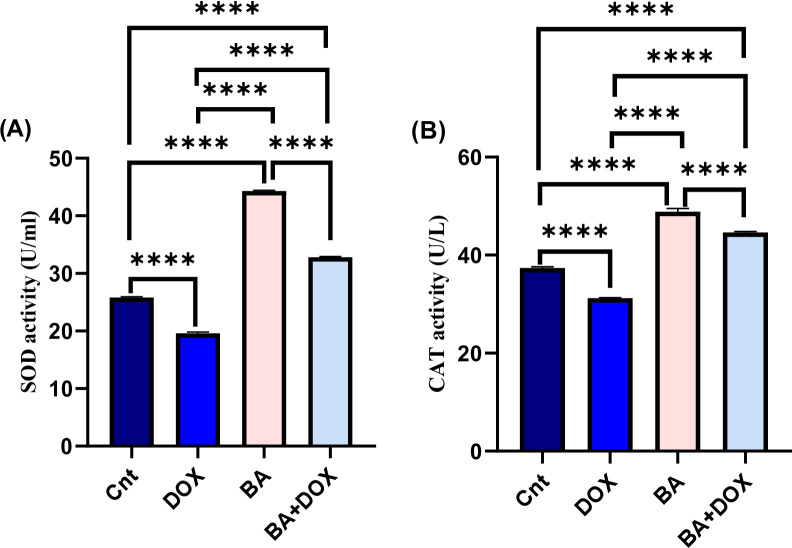
Effects of betulinic acid (BA) and doxorubicin (DOX) treatments with IC50s (64.3 and 32.9 µM, respectively) for 48 h on SOD activity (U/ml) and CAT activity (U/L) of MDA-MB-231 cells (**A**-**B**), either separately or in combination. The data provided are mean ± SEM of three independent experiments (*n* = 3) each performed in triplicate. Statistical analysis was performed using one-way ANOVA followed by Tukey’s post hoc test. Means within columns carrying **** are significantly different at (p ˂ 0.0001).

The results in Fig. 5A and B exhibited a remarkable (P ˂ 0.05) increase in the SOD and CAT activities (approximately 58.2% and 76.4%, respectively) after treatment with BA (64.3 µM) for 48 h, while the DOX treatment (32.9 µM) after 48 h exposure caused a significant decreased (approximately 75.9% and 83.4%, respectively) in relation to the control, although the BA + DOX treated cells had an increase in SOD and CAT activities relative to that of the control group.

### Molecular docking study

Molecular docking of BA and DOX against HIF1A (8HE3-A), PDCD4 (2RG8-B), PTEN (1D5R-A) and SMAD7 (2DJY-A) are represented in Table 3. BA showed the highest binding affinity (pKi: 6.6) and (pKi: 6.75) toward PDCD4 and PTEN, respectively.

**Table 3:** The molecular docking of BA and DOX with HIF1A, PDCD4, PTEN and SMAD7.

Compound	HIF1A (target site PDB ID: 8HE3-A)			PDCD4 (target site PDB ID: 2RG8-B)			PTEN (target site PDB ID: 1D5R-A)			SMAD7 (target site PDB ID: 2DJY-A)		
	Binding Free Energy (kcal/mol)	pKi	Ligand Efficiency (kcal/mol/non-H atom)	Binding Free Energy (kcal/mol)	pKi	Ligand Efficiency (kcal/mol/non-H atom)	Binding Free Energy (kcal/mol)	pKi	Ligand Efficiency (kcal/mol/non-H atom)	Binding Free Energy (kcal/mol)	pKi	Ligand Efficiency (kcal/mol/non-H atom)
Betulinic acid	-6.8	4.99	0.2061	-9.0	6.6	0.2727	-9.2	6.75	0.2788	-6.3	4.62	0.1909
Doxorubicin	-6.8	4.99	0.1744	-7.8	5.72	0.2	-7.3	5.35	0.1872	-6.8	4.99	0.1744

**Fig. 6:**
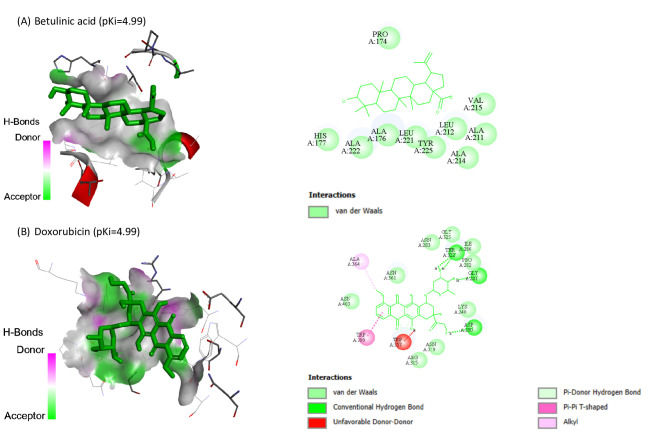
Co-crystallized ligands redocked in Hypoxia-Inducible Factor 1-alpha (HIF-1α), with mapping surface showing betulinic acid (BA), and doxorubicin (DOX) occupying the active pocket of HIF-1α. The binding modes and interactions were predicted using InstaDock software. The best docking poses were selected based on binding affinity scores. The docking results are presented as predictive and hypothesis generating.

**Fig. 7:**
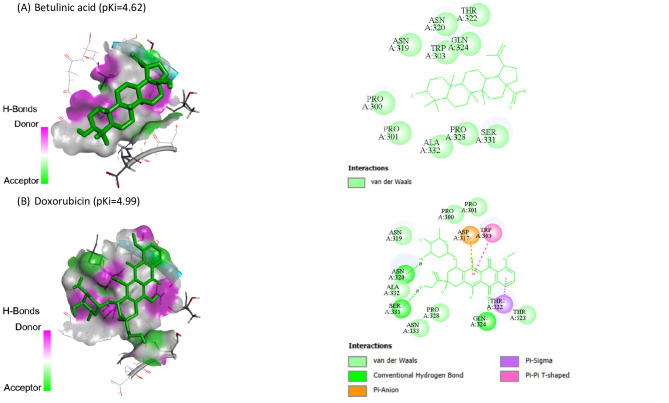
Co-crystallized ligands redocked in SMAD7, with mapping surface showing betulinic acid (BA), and doxorubicin (DOX) occupying the active pocket of SMAD7. The binding modes and interactions were predicted using InstaDock software. The best docking poses were selected based on binding affinity scores. The docking results are presented as predictive and hypothesis generating.

**Fig. 8:**
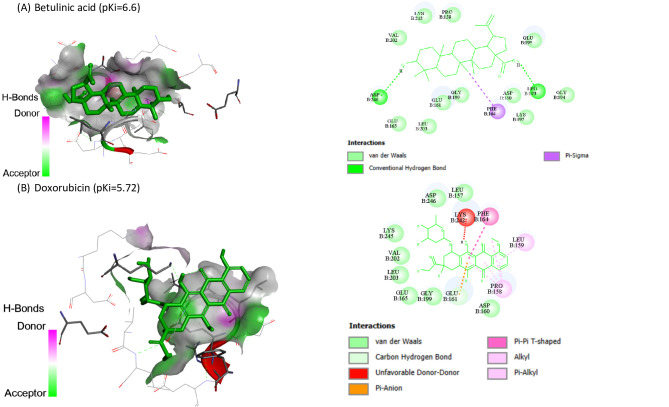
Co-crystallized ligands redocked in Programmed Cell Death 4 (PDCD4), with mapping surface showing betulinic acid (BA), and doxorubicin (DOX) occupying the active pocket of PDCD4. The binding modes and interactions were predicted using InstaDock software. The best docking poses were selected based on binding affinity scores. The docking results are presented as predictive and hypothesis generating.

**Fig. 9:**
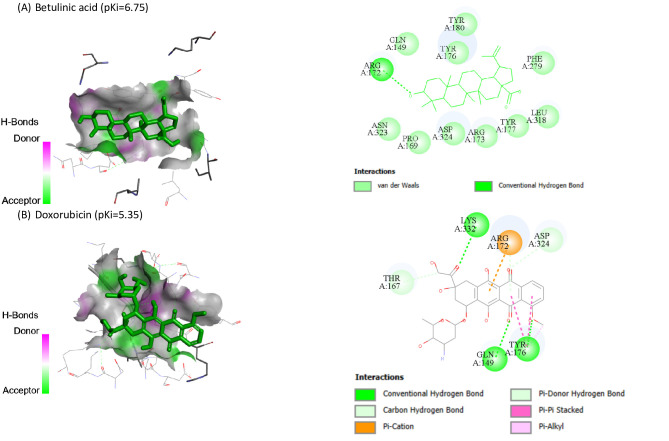
Co-crystallized ligands redocked in Phosphatase and tensin homolog (PTEN), with mapping surface showing betulinic acid (BA), and doxorubicin (DOX) occupying the active pocket of PTEN. The binding modes and interactions were predicted using InstaDock software. The best docking poses were selected based on binding affinity scores. The docking results are presented as predictive and hypothesis generating.

Also, BA and DOX exhibited pKi of 4.99 toward HIF1A, while BA exhibited the lowest affinities (pKi: 4.62). Co-crystallized ligands redocked in HIF1A, SMAD7, PDCD4, and PTEN are shown in Figs. 6, 7, 8 and 9, respectively.

## Discussion

An anthracycline called doxorubicin is frequently used to treat and control a number of cancers, such as testicular, breast, and lung cancer^30^. Additionally, this medication is generally regarded as the most effective treatment for triple-negative breast cancer (TNBC). However, DOX is known to produce cardiac adverse effects in certain people, including acute coronary syndromes, heart failure, and altered ECGs^31^.

Therefore, researching novel natural compounds that may improve the benefits of DOX and potentially reduce its toxicity is one way to lessen its toxicity when treating patients with TNBC^32^.

By altering crucial signaling pathways in TNBC, BA is a well-known natural substance that can cause apoptosis and prevent tumorigenesis^33^. Additionally, BA can cause cancer cells to produce more reactive oxygen species (ROS), which can result in severe oxidative stress^34^.

To the best of our knowledge, however, no research has been done on the molecular targets suggesting such effect. In the current study, we use breast cancer cell line (MDA-MB-231) as a well-established model of triple negative breast cancer with its aggressive behavior and limited therapeutic effect.

In the current study, we showed that treatment with BA and/or DOX was associated with anti-proliferative and cytotoxic effects in MDA-MB-231 cells with variable potency under the tested conditions. MDA-MB-231 cells were more responsive to DOX since the compound showed lower IC50 value compared with BA. This finding agrees with the fact that DOX is prescribed mainly in the treatment of triple-negative breast cancer^35^. Preliminary observations indicated that 48 h produced more pronounced effects compared to shorter exposure time. BA treatment showed anti-proliferative effect that agrees with previous studies which investigated a decrease in proliferation of MDA-MB-231 cells after treatment with BA organic salts^36^. Another study reported that BA suppressed proliferation of the SKOV3 and SW626 ovarian cell lines in a dose-dependent manner^37^.

Many recent reports, including the current study, indicated promising chemo-modulatory action of BA when combined with other chemotherapeutic agents used against various cancers^38^.

The strong anticancer activity of BA on cancer cells contrasts with its apparent lack of cytotoxicity on nonmalignant cells. Therefore, it has been noted that non-transformed cells of various origins, such as fibroblasts, melanocytes, neuronal cells, and peripheral blood lymphocytes, are significantly more resistant to the cytotoxic action of BA than cancer cells^39^. DOX well-established dose-dependent cardiotoxicity is attributed to its known cytotoxic effects on both malignant and non-cancerous cells^40^. This distinction emphasizes the potential benefit of mixing BA with traditional chemotherapeutic drugs to improve anticancer activity while perhaps lowering side effects.

Furthermore, we have investigated the potential changes associated with this anti-proliferative effect, such as modulation of apoptosis, necrosis, and genetic modifications, as no formal interaction analysis was performed.

We performed Annexin V/PI staining assay to investigate whether BA suppressed breast cancer cells proliferation by inducing apoptosis. Many reports have attributed the anticancer effects of BA; we have examined the apoptotic and necrotic cell death after individual and combined BA, and DOX treatment. Our results indicated that BA was associated with apoptotic features in breast cancer cells at the concentration corresponded to its IC50 value. In MDA-MB-231 cells, apoptotic cell death was prevalent in BA single treatment. Interestingly, a combination of BA with DOX increased the population of necrotic cells by 2.5 folds compared to control.

Such data might be important in future combination therapy studies aiming to bypass apoptosis as a major cell death mechanism in DOX-resistant cancer cells.

It is widely reported that BA exhibits apoptotic induction effect on various cancer cells. Wang et al. showed that BA induces apoptosis of gallbladder cancer cells via repressing SCD1^41^. Liu’s study demonstrated that BA induces autophagy-mediated apoptosis through suppression of the PI3K/AKT/mTOR signaling pathway and inhibits hepatocellular carcinoma^42^. Shen also verified that BA induces ROS-dependent apoptosis and S-phase arrest by inhibiting the NF- κB pathway in human multiple myeloma^42^.

Numerous clinical conditions, such as inflammation, cardiovascular disease, cancer, neurological diseases, and even aging, are believed to be significantly influenced by oxidative stress. Moreover, oxidative stress contributes to a number of alterations in cell structure and function as well as DNA mutations that lead to cancer. Antioxidants may have a crucial role in preventing the onset of diseases like cancer^43^. Research and investigations have shown that BA offers promising antioxidant effects in the battle against oxidative stress^44^.

It’s interesting to note that BA increased the activity of antioxidant enzymes like SOD and CAT, indicating a possible function in regulating cellular redox balance. Contrarily, DOX decreased antioxidant defenses, which is in line with its well-known pro-oxidant properties. These results suggest that BA may have anticancer effects through mechanisms other than oxidative stress induction, such as redox regulation and alternate apoptotic pathways. The current research indicates that BA may control redox homeostasis instead of merely causing oxidative damage, despite the fact that oxidative stress has been linked to cancer cell death. Therefore, the relationship between antioxidant enzyme activity and cytotoxicity may be complex and context-dependent.

About 60% of human genes have their expression regulated by miRNA molecules, which are small, non-coding, single-stranded RNAs^45^. The activity of one or more genes can be concurrently regulated by distinct miRNAs. MiRNA molecules may play a significant role in the development of breast cancer, according to earlier research. By targeting tumor suppressor genes, they can increase invasion and metastasis while downregulating miRNAs crucial for preserving physiological state^46^.

miR-21 regulates breast cancer cell invasion and migration. To investigate the roles of miR21 in regulating breast cancer cell invasion and migration, we studied its molecular effect of genes such as PTEN, SMAD7, HIF1A, and PDCD4. After treatment with BA for 48 h, the miR-21 level was downregulated as compared to control followed by combined treatment with BA + DOX.

Breast, liver, prostate, colorectal, cervical, and pancreatic cancers are among the many tumors against which BA has demonstrated anticancer properties^47^. The bioactivities of BA have been linked to a number of targets, including NF-κB, P53, PI3K, ERBB2, STAT3, ERα, and HIF-1α.

A protein called Hypoxia-Inducible Factor 1-alpha (HIF-1α) functions as a transcription factor, assisting cells in responding to low oxygen (hypoxia) by activating particular genes that enable survival and adaptation. By activating genes including VEGF (vascular growth), EPO (red blood cell production), and MMPs (matrix metalloproteinases), which encourage cell migration and tissue invasion, HIF-1α plays a role in the onset and spread of breast cancer^48^. Our study showed that BA suppressed the expression of HIF-1α followed by combined treatment with BA + DOX as compared to control modulating cancer treatment.

A crucial intracellular modulator of the Transforming Growth Factor-beta (TGF-β) signaling pathway, SMAD7 belongs to the SMAD family of proteins. Its expression and activity vary greatly between normal and malignant tissues and may be influenced by the tumor setting as well as the balance of other signaling pathways^49^. In our study, treatment with BA + DOX showed the lowest expression level of SMAD7 in breast cancer cell line followed by treatment with BA.

The tumor suppressor gene PDCD4 (Programmed Cell Death 4) is essential for controlling cell division, apoptosis, and protein translation. It was initially discovered as a gene that was activated during programmed cell death. Since then, it has been found as a powerful tumor suppressor that is often downregulated in malignancies of the breast, lung, colon, stomach, and pancreas^50^. After treatment with DOX for 48 h followed by BA, the expression of PDCD4 was increased indicating the potent effect of BA in treatment of lung cancer.

The tumor suppressor gene phosphatase and tensin homolog (PTEN) regulates the PI3K/AKT signaling pathway and is essential for preserving the equilibrium between cell death and survival. Uncontrolled activation of the PI3K/AKT pathway results from the loss or mutation of PTEN, which eliminates its regulatory impact. Cellular expansion, resistance to apoptosis, and the cells’ capacity to avoid regular regulatory processes are the outcomes of this. Loss of PTEN is frequently linked to enhanced angiogenesis, metastasis, and cell migration—all of which aid in the growth and spread of malignancies^51^. DOX treatment for 48 h showed the highest expression of PTEN then BA.

Our in silico study predicted the interaction of BA with HIF1A, PDCD4, PTEN and SMAD7. The molecular docking results suggested potential binding interactions of BA with PTEN and PDCD4 followed by DOX. On the other hand, DOX showed potential binding interactions with SMAD7 than BA. BA and DOX indicated the same possible interaction with HIF1A.

Despite the fact that molecular docking study revealed putative interactions between BA and the targets under investigation, these conclusions are predicated on computational predictions, these findings are predictive and do not confirm biological effects or functional relevance. As a result, it is appropriate to interpret the docking results as generating hypotheses.

Betulinic acid’s pleiotropic nature—a feature exhibited with several natural compounds—may account for its capacity to interact with a variety of molecular targets. These substances frequently regulate several signaling pathways at once rather than just one particular protein, producing coordinated biological effects. The observed alterations in apoptosis, redox balance, and gene expression could be attributed to this multi-target behavior.

According to previous studies, BA can affect a variety of cellular processes, such as oxidative stress reactions, apoptotic control, and mitochondrial function^52,53^. This provides credibility to the concept that a network-based mechanism, instead of a single target interaction, may be responsible for its anticancer effects.

## Conclusion

The present study showed that BA acts as suppressor for miR-21 in triple-negative breast cancer cells. In our study, we studied cell toxicity, apoptosis, and necrosis induction, as well as its influence on miR-21 related pathways that reduce HIF1A and SMAD7 and increase PDCD4 and PTEN expression. Significantly, at the tested concentrations, the combination treatment showed differences in biological responses compared to single treatments; however, no formal interaction analysis was performed. Additionally, the molecular docking results suggested potential interactions with selected targets; however, these findings are predictive and hypothesis-generating, and do not confirm biological mechanisms. To confirm these findings and clarify the underlying mechanisms, more research is needed.

## Data Availability

All data generated or analyzed during this study are included in this published article and its supplementary information files.

## Abbreviations

BA: Betulinic acid
BC: Breast cancer
CAT: Catalase
DOX: Doxorubicin
HIF-1α: Hypoxia-Inducible Factor 1-alpha
PDCD4: Programmed Cell Death 4
PTEN: Phosphatase and tensin homolog
ROS: Reactive oxygen species
SOD: Superoxide Dismutase
TNBC: Triple-negative breast cancer
